# Mutual stimulatory signaling between human myogenic cells and rat cerebellar neurons

**DOI:** 10.14814/phy2.15077

**Published:** 2021-10-29

**Authors:** Michal Tamáš, Stanislava Pankratova, Peter Schjerling, Casper Soendenbroe, Ching‐Yan Chloé Yeung, Cristian Pablo Pennisi, Jens R. Jakobsen, Michael R. Krogsgaard, Michael Kjaer, Abigail L. Mackey

**Affiliations:** ^1^ Institute of Sports Medicine Copenhagen Department of Orthopaedic Surgery Copenhagen University Hospital – Bispebjerg and Frederiksberg Copenhagen Denmark; ^2^ Center for Healthy Aging Department of Clinical Medicine University of Copenhagen Copenhagen Denmark; ^3^ Laboratory of Neural Plasticity Department of Neuroscience University of Copenhagen Copenhagen Denmark; ^4^ Comparative Pediatrics and Nutrition Department of Veterinary and Animal Sciences University of Copenhagen Frederiksberg C Denmark; ^5^ Xlab Department of Biomedical Sciences Faculty of Health and Medical Sciences University of Copenhagen Copenhagen Denmark; ^6^ Regenerative Medicine Group Department of Health Science and Technology Aalborg University Aalborg Denmark; ^7^ Section for Sports Traumatology M51 Department of Orthopaedic Surgery Copenhagen University Hospital – Bispebjerg and Frederiksberg Copenhagen Denmark

**Keywords:** acetylcholine receptor, innervation, myogenesis, neuron, skeletal muscle

## Abstract

Insight into the bidirectional signaling between primary human myogenic cells and neurons is lacking. For this purpose, human myogenic cells were derived from the semitendinosus and gracilis muscles of five healthy individuals and co‐cultured with cerebellar granule neurons from two litters of 7‐day‐old Wistar rat pups, in muscle medium or neural medium, alongside monocultures of myogenic cells or neurons. RT‐PCR was performed to determine human mRNA levels of GAPDH, Ki67, myogenin, and MUSK, and the acetylcholine receptor subtypes CHRNA1, CHRNB1, CHRNG, CHRND, and CHRNE, and rat mRNA levels of GAPDH, Fth1, Rack1, vimentin, Cdh13, and Ppp1r1a. Immunocytochemistry was used to evaluate neurite outgrowth (GAP43) in the presence and absence of myogenic cells. Co‐culture with primary neurons lead to higher myogenic cell gene expression levels of GAPDH, myogenin, MUSK, CHRNA1, CHRNG, and CHRND, compared to myogenic cells cultured alone. It appeared that neurons preferentially attached to myotubes and that neurite outgrowth was enhanced when neurons were cultured with myogenic cells compared to monoculture. In neural medium, rat mRNA levels of GAPDH, vimentin, Cdh13, and Ppp1r1a were greater in co‐culture, versus monoculture, whereas in muscle medium co‐culture lead to lower levels of Fth1, Rack1, vimentin, and Cdh13 than monoculture. These findings demonstrate mutually beneficial stimulatory signaling between rat cerebellar granule neurons and human myogenic cells, providing support for an active role for both the neuron and the muscle cell in stimulating neurite growth and myogenesis. Bidirectional muscle nerve signaling.

## Graphical abstract

These findings demonstrate mutually beneficial stimulatory signaling between rat cerebellar granule neurons and human myogenic cells. The presence of myogenic cells stimulated neurite growth. Species specific PCR of the mono‐cultures vs. co‐cultures revealed effects of co‐culture and/or medium type (statistical main effects and interactions indicated).

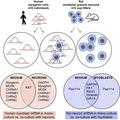

## 1 INTRODUCTION

The interplay between the skeletal muscle and the central nervous system is multifaceted. Skeletal muscle fibers are fully reliant on neural input, not only for contraction to facilitate movement but also for survival. The single point of contact between motoneuron and myofiber, the neuromuscular junction (NMJ), is well characterized and the molecular basis for NMJ development has been extensively studied (Liu & Chakkalakal, 2018; Missias et al., 1996; Sanes & Lichtman, 2001). The initiation of an action potential in the sarcolemma is the primary role of the motoneuron in relation to the myofibers it innervates. However there is evidence that communication travels in both directions across the NMJ, with the myofiber transmitting molecular signals to the motoneuron in a so‐called retrograde fashion (Chakkalakal et al., 2010; Mills et al., 2018; Zahavi et al., 2015), in a similar manner to the uptake of the tetanus neurotoxin at the NMJ (Schiavo et al., 2000). Maintenance of the NMJ is therefore likely to be dependent on activity on both the muscle and motoneuron sides of the NMJ (Soendenbroe et al., 2021). In addition to motoneurons, cerebellar neurons are influenced by factors produced by exercising muscle. For example, myofiber‐derived vascular endothelial growth factor (VEGF) has been shown to induce hippocampal neurogenesis in mice (Rich et al., 2017). In humans, exercise and breaks in sitting during the day have been shown to improve working memory and executive function (Wheeler et al., 2020), and exercise training has been reported to result in increased hippocampal volume and improved memory (Erickson et al., 2011), illustrating the importance of skeletal muscle for neurons throughout the central nervous system. In principle, muscle‐derived factors could influence cerebellar neurons through retrograde signaling via the NMJ, as well as by crossing the blood–brain barrier from the circulation. To our knowledge, bidirectional signaling between human myogenic cells and neurons has not yet been investigated in a systematic manner with quantitative data. The implications of two‐way muscle nerve activity are wide‐ranging, including gaining mechanistic insight into neuromuscular diseases and the role of exercise in preserving physical and cognitive function with aging.

Cell culture represents a useful tool in the study of cell–cell communication, although it seems fundamental differences exist concerning the formation of postsynaptic NMJ components in cell cultures of myogenic cells of animal versus human origin (Mis et al., 2017). For example, it appears that human myotubes are slower than animal to develop a basement membrane (Askanas et al., 1987), and that clustering of acetylcholine receptors readily occurs on myotubes of animal origin even in the absence of neurons (Sytkowski et al., 1973), whereas this is not the case for human myotubes, as discussed elsewhere (Mis et al., 2017). For study of the NMJ specifically, some elegant cell culture models have been established with human skeletal muscle cells and rodent neurons (Saini et al., 2019, 2020), or human neurons (Guo et al., 2011; Steinbeck et al., 2016), but quantitative data from purified populations of primary human myogenic cells cultured with neurons are lacking. One of the main challenges is obtaining sufficient numbers of purified motoneurons for experiments with biological and technical replicates, where up to 5 million neurons might be required (and it is estimated that only 1% of all cells in the spinal cord of rat embryos are motoneurons (Jacquier et al., 2019)). Cerebellar granule neurons (CGNs) represent a more homogenous, and plentiful, cell population (Bilimoria & Bonni, 2008) and, when co‐cultured with myogenic cells, can provide insight into the potential of myogenic and neural cells to stimulate cell growth in a mutual manner.

One of the initial and key features of neurons in cell culture is neurite outgrowth, which is visible under light microscopy 24 h after plating. A recent study mapping the subcellular transcriptomes of neuron growth cones versus their parent cell bodies reported that mRNA for genes such as Fth1, Rack1, vimentin, Cdh13, and Ppp1r1a are enriched in growth cones and therefore represent useful markers of early stage neural growth (Poulopoulos et al., 2019). In relation to myogenic cells, in addition to the classic myogenic marker of differentiation myogenin and Ki67 as a general cell proliferation marker, it would appear that the acetylcholine receptor subunits and MuSK, key molecules for establishing the NMJ, are expressed in primary myogenic cells isolated from healthy human skeletal (Soendenbroe et al., 2020). Importantly, this occurs in the absence of neurons and it is possible the presence of neurons would further stimulate transcription of these genes. The main aim of this study was to determine the reciprocal influence of primary human myogenic cells and rat cerebellar neurons in cell culture.

## 2 METHODS

For the human participants, eight individuals, aged 18–50, scheduled for reconstructive surgery after anterior cruciate ligament (ACL) rupture, gave informed consent to donate excess muscle tissue. Exclusion criteria included diabetes, arthritis, and obesity. The study was approved by the Committee on Health Research Ethics for the Capital Region of Denmark (Ref. No. H‐3‐2010‐070) and was conducted according to the Declaration of Helsinki.

### 2.1 Human tissue collection and myogenic cell culture

Muscle tissue was collected from patients scheduled for ACL surgery (Bechshoft, Schjerling et al., 2019), where the semitendinosus and gracilis tendons are used as grafts to reconstruct the torn ACL. When the waste muscle and tendon tissue were set aside for this experiment, it was immediately transferred into PBS containing 1% penicillin/streptomycin (P4333, Sigma‐Aldrich) and processed under standard cell culture conditions, as described in detail (Bechshoft, Schjerling et al., 2019). Approximately 500–800 mg of muscle tissue was minced, and incubated in sterile filtered digestion medium (C‐23260, PromoCell; 11088815001, Roche Diagnostics A/S, Denmark; D4593‐1G, Sigma‐Aldrich) for 1 h at 37°C, 5% CO_2_ (Forma Steri‐cycle CO2 Incubator 317, Thermo Fisher Scientific, Denmark), with trituration every 15 min. After digestion, 5 ml of culture medium (C‐23060, PromoCell; C‐39365, PromoCell; G6784, Sigma; ALB – S1810, Biowest), containing 15% FCS and 1% L‐Glutamine–Penicillin–Streptomycin, was added and the suspension was filtered through a 100 μm pore sized cell strainer (352360, Falcon). After centrifugation (600 *g* for 6 min at room temperature––and for all subsequent centrifugation steps), the supernatant was discarded and the pellet was carefully resuspended in 1 ml of culture medium and transferred to a T25 flask (690170, Greiner Bio‐One). The medium was changed every 2 days. On the first change in medium, old medium was centrifuged and the pellet was dissolved in 1 ml of new culture medium and returned to the flask. The cells were cultured for 5–7 days (approximately 80% confluency).

### 2.2 Myogenic cell purification

After washing the cells two times with sterile PBS, 0.05% trypsin‐EDTA solution (03‐054‐1B, Biological Industries) was added. To prevent damage to the CD56 antigen on the surface of the myogenic cells, the time of trypsinization was limited to the minimum required for cell detachment (<2 min). The detached cells were suspended in culture medium and centrifuged. The pellet was resuspended in 10 ml of PBS and centrifuged again. The pellet was resuspended in 170 μl of MACS buffer (130‐091‐221, Miltenyi Biotec Norden AB) and 35 μl of CD56 magnetic beads (130‐050‐401, Miltenyi Biotec Norden AB), and incubated for 15 min at 5°C. After addition of 5 ml of MACS buffer, cells were centrifuged and the pellet was homogenized in 1 ml of MACS buffer before being loaded to a large cell column (130‐042‐202, Miltenyi Biotec Norden AB) with a 30 μm pre‐separation filter (130‐041‐199 407, Miltenyi Biotec Norden AB). The cell column was fixed to a MiniMACS separator (130‐090‐312, Miltenyi Biotec Norden AB) attached to a MultiStand magnet (130‐090‐312, Miltenyi Biotec Norden AB). The column was equilibrated by 500 μl of MACS buffer prior to adding the cell suspension. As the cell suspension passes through the column, CD56‐positive (myogenic cells) are retained by the magnet while CD56‐negative cells run freely through. To release the CD56‐positive cells, the pre‐separation filter was removed and 2.5 ml of MACS buffer was flushed through the column with a piston. Culture medium (5ml) was added before centrifugation. Cells were then counted in Neubauer counting chamber (0.0025 mm^2^, depth 0.100 mm, cat. no. 1300000, Labor Optik, Lancing, UK) and frozen or plated immediately. Plated cells were between passages 2 and 5. From the purified pool of myogenic cells, 20,000 were plated (5000 cells/cm^2^) per well of a 12‐well plate (665180, Greiner Bio‐One) containing an 18 mm sterilized glass coverslip (0111580, Marienfeld) and 1 ml of culture medium. Primary human myogenic cell cultures from eight patients were established in this manner in this study (three patients for myogenic cell purity evaluation, five patients for co‐culture). Given that the surgeries, where the tissue was collected, were on different days, the cells from most of the donors were frozen after sorting. The cells from five patients used for the co‐culture experiments were cultured in parallel. Myogenic cell purity evaluation was performed on cell cultures prior to the co‐culture experiments, by the same researcher performing all the cell cultures (including the cell sorting).

### 2.3 Rat neuron isolation

Wistar rats were obtained from Charles River (Sulzfeld, Germany) and handled in accordance with the Danish Animal Welfare Act and approved by the Department of Experimental Medicine at the University of Copenhagen (P 20‐070). Upon arrival, rat dams with pups were housed in standard polycarbonate cages and kept under climate‐controlled housing conditions with a 12‐h light cycle and free access to water and rat chow. Cerebellar granule neurons (CGNs) were isolated from 7‐day‐old Wistar rat pups, sacrificed by cervical dislocation, as recently described (Dmytriyeva et al., 2020). Briefly, the cerebella were resected, cleared from meninges, and chopped in ice‐cold Krebs buffer. Hereafter, the tissue was digested with trypsin (cat. no. T0303‐1G, Sigma‐Aldrich), cell pellet was washed in DNAse I (Sigma‐Aldrich) and soybean trypsin inhibitor (Sigma‐Aldrich). After centrifugation, the cells were resuspended in Neurobasal^TM^‐A medium (10888022, Invitrogen) supplemented with 2% (v/v) B27 (17504044, Gibco), 1% (v/v) GlutaMax (35050061, Gibco), 100 U/ml penicillin–streptomycin (15070063, Gibco), 20 mM HEPES (15630080, Gibco; all from Thermo Fisher Scientific), hereafter called neural medium. CGNs were isolated from two litters of rats, on the same day, where cerebelli from 2 to 3 pups from each litter were merged and purified simultaneously.

### 2.4 Co‐culture of human myogenic cells and rat neurons

**FIGURE 1:**
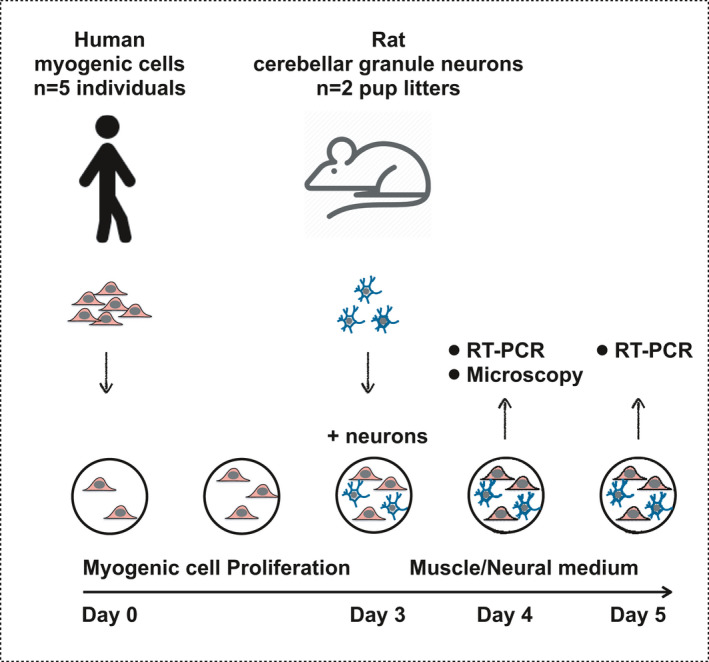
Overview of study design. Purified human myogenic cells from healthy individuals were cultured on glass coverslips under proliferation conditions for 3 days. Rat cerebellar granule neurons were added and the co‐cultures were evaluated after 1 or 2 days

An overview of the time course and study design is illustrated in Figure 1. Human myogenic cells were cultured to proliferate alone for 3 days. Then the medium was changed to either “neural medium,” or differentiation “muscle medium” (Skeletal muscle basal medium, cat. no. C‐23260, PromoCell) and L‐Glutamine–Penicillin–Streptomycin solution, and 20,000 CGNs were added to each well. One set of cells was co‐cultured for 1 day and another set for 2 days. Monocultures (myogenic cells alone, or CGNs alone) were carried out in parallel. Six replicates of each condition were prepared in order to have triplicates for immunocytochemistry and triplicates for qPCR.

### 2.5 Immunocytochemistry (ICC)

Cells for ICC were washed with PBS and fixed with Histofix (cat. no. 01000, Histolab). Cells cultured alone were fixed for 8 min while the co‐cultures were fixed for 20–30 min (for improved adherence to the coverslip). Histofix was aspirated from the wells and cells were washed three times with PBS and then stored in PBS at 4°C until ICC staining. Cells were permeabilized with 500 μl of 0.01% Triton‐X solution (9002‐93‐1, Sigma‐Aldrich) in 0.05 M Tris‐buffered saline (TBS) for 8 min. Primary antibodies (see Table 1) in blocking buffer (1% Bovine Serum Albumin; cat. no. A3912‐100G, Sigma‐Aldrich), containing 0.1% sodium azide (cat. no. S‐304 2002, Sigma‐Aldrich) in TBS were added and incubated overnight at 4°C. After washing with TBS, the cells were incubated with secondary antibodies and alpha‐bungarotoxin (α‐BTX) (see Table 1) in blocking buffer for 1 h at room temperature. After washing, the coverslips were mounted on a glass slides with a drop of Prolong Gold Antifade reagent containing DAPI (cat. no. P36931, Invitrogen). The combination of antibodies for the main outcome of neural outgrowth was mouse anti‐desmin (cat. no. ab8470) as a myogenic cell marker, rabbit anti‐GAP43 as a marker for developing neurons and growth cones, and aBTX for the detection of neuromuscular junctions (NMJ). For the cell purity tests, rabbit anti‐desmin (cat. no. ab32362) and mouse anti‐TE7 (fibroblast marker; cat. no. CBL271) were used.

**TABLE 1:** Antibodies and toxins used for immunocytochemical staining

	Host species	Target	Dilution	Company	Cat. no.
Co‐culture					
Primary Ab	Mouse	Desmin	1:100	Abcam	ab8470
Primary Ab	Rabbit	GAP43	1:1000	Millipore	AB5220
Secondary Ab	Goat 568 (red)	anti‐rabbit	1:500	Invitrogen	A11036
Secondary Ab	Goat 680 (far red)	anti‐mouse	1:100	Invitrogen	A21057
Toxin	aBTX 488 (green)	NMJ	1:100	Invitrogen	B13422
Myogenic purity					
Primary Ab	Rabbit	Desmin	1:1000	Abcam	ab32362
Primary Ab	Mouse	TE7	1:100	Millipore	CBL271
Secondary Ab	Goat 488 (green)	anti‐mouse	1:500	Invitrogen	A11029
Secondary Ab	Goat 568 (red)	anti‐rabbit	1:500	Invitrogen	A11036

### 2.6 Myogenic cell purity microscopy

Cell purity was evaluated on myogenic cells cultured alone in proliferation medium for 3 days, followed by muscle medium for 4 days. Myogenic cell purity was determined from desmin‐TE7 images. Images were captured using an Olympus BX51 microscope, and a digital camera (Olympus DP71; Olympus Deutschland, Hamburg, Germany) controlled by the software Olympus cellSens Standard 1.14 (Olympus Soft Imaging Solutions, Münster, Germany). Myogenic cell purity was determined by counting the number of desmin + cells and expressing this number as a percentage of all cells counted.

### 2.7 Neuron image acquisition and processing

The coverslips were imaged in a slide scanner (Zeiss AxioScan.Z1, Slide Scanner, Germany) using a Plan Apochromat 20×/0.8 NA objective. CCD camera recorded DAPI (blue), aBTX (green), GAP43 (red), and desmin (far‐red) at 6.45 μm/pixel with automatic stitching of the tiles. All images were analyzed with ImageJ software version 1.5p (NIH) using two macros. First, for each channel the background was subtracted by setting up a rolling ball radius to 50 pixels. Then enhanced contrast function was applied and saturated pixels were set to 2%. Finally minimum and maximum gray values for the pixels were set to 30 and 300, respectively, and the image was saved in jpg format. Next, the second replicate in neural medium from each myoblast donor was chosen for image analysis of both culture conditions––neurons only and neurons + myoblasts. For patients #1 and #4, the third replicate was used because of poor quality of the original image. Using a second macro, the DAPI channel was opened and transformed to grayscale, while the other channels were hidden. A neural nucleus was randomly selected and a 199 × 199 pixel RGB image around the chosen nucleus, including the green and red channels, was automatically created. This was repeated for 10 nuclei per image. The neural nuclei are smaller and more intensely stained with DAPI than the myoblast nuclei and are thus clearly distinguishable from the myoblasts based on DAPI staining alone.

### 2.8 mRNA extraction

Coverslips assigned to PCR analysis were moved to another plate and 1 ml of TRI Reagent (cat. no. TR118, Molecular Research Center Inc.) was added to each well and resuspended 20 times. The suspension was then moved to a 2 ml sterile Biospec tube (5225, Bio Spec Products Inc.) and stored at –80°C until further processing. For extraction of RNA, 100 μl of bromo‐chloropropane was added to the TRI Reagent and left at room temperature for 15 min and then spun at 12,000 *g* for 15 min in order to separate the sample into an aqueous phase (containing RNA) and an organic phase. To precipitate the RNA from the aqueous phase (350 μl), 80 μg of glycogen (cat. no. 10814‐010, Invitrogen) and 350 μl of isopropanol were added, mixed, and left for 10 min at room temperature. Then the sample was spun at 12,000 *g* for 8 min at room temperature. The RNA pellet was washed with 1 ml of 75% ethanol, spun twice at 7500 *g* for 5 min, removing the supernatant between spins, and dissolved in 100 μl of RNase‐free water. Then re‐precipitated with 10 μl of 3 M sodium acetate, pH 5.5 and 200 μl of 99% ethanol applying the same washing procedure as above. Finally, the pellet was dissolved in 10 μl of RNase‐free water. Total RNA concentration and purity were determined by spectroscopy with RiboGreen RNA assay Kit from 1 μl of RNA (R11490, Thermo Fisher).

### 2.9 RT‐PCR

First, one hundred nanograms of RNA was converted to 20 μl cDNA using Omniscript reverse transcriptase (Qiagen) and 1 mM poly‐dT according to the manufacturer's protocol (Qiagen). Next, 0.25 μl cDNA was amplified in a 25 μl of SYBR Green polymerase chain reaction (PCR) containing 1× Quantitect SYBR Green Master Mix (Qiagen) and 100 nM of each primer for every target mRNA (see Table 2). The rat targets were selected based on a report of their enrichment in neurites compared to neuron cell bodies (Poulopoulos et al., 2019). A MX3005P real‐time PCR machine (Stratagene) was used for monitoring the amplification, and a standard curve was made with known concentrations of the cloned PCR products or DNA oligonucleotides (Ultramer oligos, Integrated DNA Technologies, Inc.) including a DNA sequence corresponding to the expected PCR product. The Ct values were related to the standard curve. Melting curve analysis after amplification was used to confirm the specificity of the PCR products and RPLP0 mRNA was chosen as an internal control. To support the use of RPLP0, another unrelated “constitutive” mRNA, GAPDH, was measured, and normalized with RPLP0. All targets were normalized to RPLP0. The geometric mean of the triplicates was determined, and then the geometric mean of the two neuron donors. Human myogenic cell mRNA data are expressed relative to the geometric mean of the respective mRNA data for the myogenic monocultures on day 4 in muscle medium.

**TABLE 2:** Primers used for PCR and their sequences

Target	Accession number and version	Sense	Anti‐sense
Human			
RPLP0	NM_053275.3	GGAAACTCTGCATTCTCGCTTCCT	CCAGGACTCGTTTGTACCCGTTG
GAPDH	NM_002046.4	CCTCCTGCACCACCAACTGCTT	GAGGGGCCATCCACAGTCTTCT
Myogenin	NM_002479.5	CTGCAGTCCAGAGTGGGGCAGT	CTGTAGGGTCAGCCGTGAGCAG
CHRNA1	NM_000079.3	GCAGAGACCATGAAGTCAGACCAGGAG	CCGATGATGCAAACAAGCATGAA
CHRNB1	NM_000747.2	TTCATCCGGAAGCCGCCAAG	CCGCAGATCAGGGGCAGACA
CHRND	NM_000751.2	CAGCTGTGGATGGGGCAAAC	GCCACTCGGTTCCAGCTGTCTT
CHRNE	NM_000080.4	TGGCAGAACTGTTCGCTTATTTTCC	TTGATGGTCTTGCCGTCGTTGT
CHRNG	NM_005199.5	GCCTGCAACCTCATTGCCTGT	ACTCGGCCCACCAGGAACCAC
MUSK	NM_005592.3	TCATGGCAGAATTTGACAACCCTAAC	GGCTTCCCGACAGCACACAC
Ki67	NM_002417.4	CGGAAGAGCTGAACAGCAACGA	GCGTCTGGAGCGCAGGGATA
Rat			
RPLP0	NM_022402.2	CCAGAGGTGCTGGACATCACAGAG	TGGAGTGAGGCACTGAGGCAAC
GAPDH	NM_017008.4	CCATTCTTCCACCTTTGATGCT	TGTTGCTGTAGCCATATTCATTGT
Fth1	NM_012848.2	GCACTGCACTTGGAAAAGAGTGTGAA	CCTGCTCATTCAGGTAATGCGTCT
Rack1	NM_130734.2	GCCACCCCAGTGTACCTCTTTG	TCACCTGCCATACACGCACCAA
Vimentin	NM_031140.1	TCCTCTGGTTGACACCCACTCC	GTTTTTATTCAAGGTCATCGTGGTGCT
Cdh13	NM_138889.2	GCCTCAGCTTGCTGCTGCTCT	GGGAGTCAAGCTTCAGATGTGTCGT
Ppp1r1a	NM_022676.3	AGACACAGGCTCAGCGTCAAGG	TGCTCCTGAGTCTTGGGTTTGG

### 2.10 Statistical analysis

RT‐PCR data were analyzed in SigmaPlot version 13.0 (Systat Software Inc) with a two‐way repeated measures ANOVA (two‐factor repetition), where the two factors were medium type (neural or muscle) and culture condition (mono‐culture or co‐culture). When a significant interaction was observed, post hoc pairwise multiple comparisons were performed using the Student–Newman–Keuls method.

## 3 RESULTS

### 3.1 Myogenic cell purity

**FIGURE 2:**
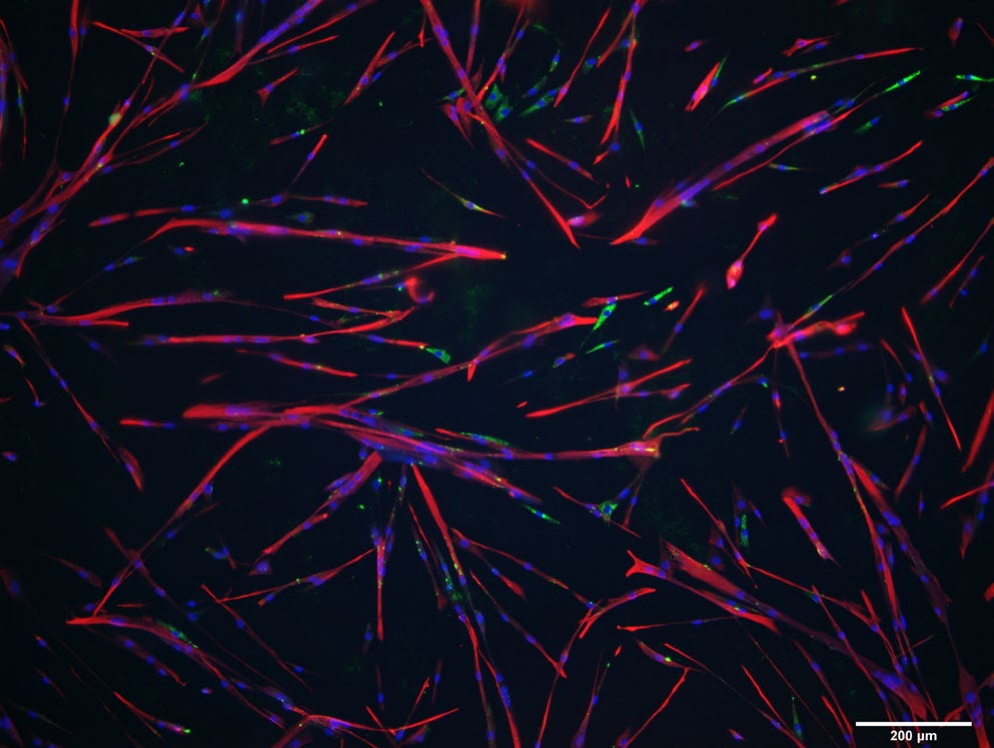
Myogenic cell purity. Immunocytochemistry of human myogenic cells cultured to proliferate for 3 days followed by 4 days of differentiation. Myogenic cells are immunoreactive for desmin (red) and non‐myogenic cells display immunoreactivity for TE7 (green, likely fibroblasts), or are unstained. DAPI stains the nuclei blue. Scale bar, 200 μm

The total number of cells evaluated from the three myogenic cell cultures were 2872, 2672, and 1404, corresponding to a myogenic cell purity of 91%, 95%, and 95%, respectively. This is in line with other studies using this method (Agley et al., 2013; Bechshoft, Schjerling et al., 2019; Bechshoft, Jensen et al., 2019). An example of one of these cell cultures is provided (Figure 2).

### 3.2 Neural cell observations

**FIGURE 3:**
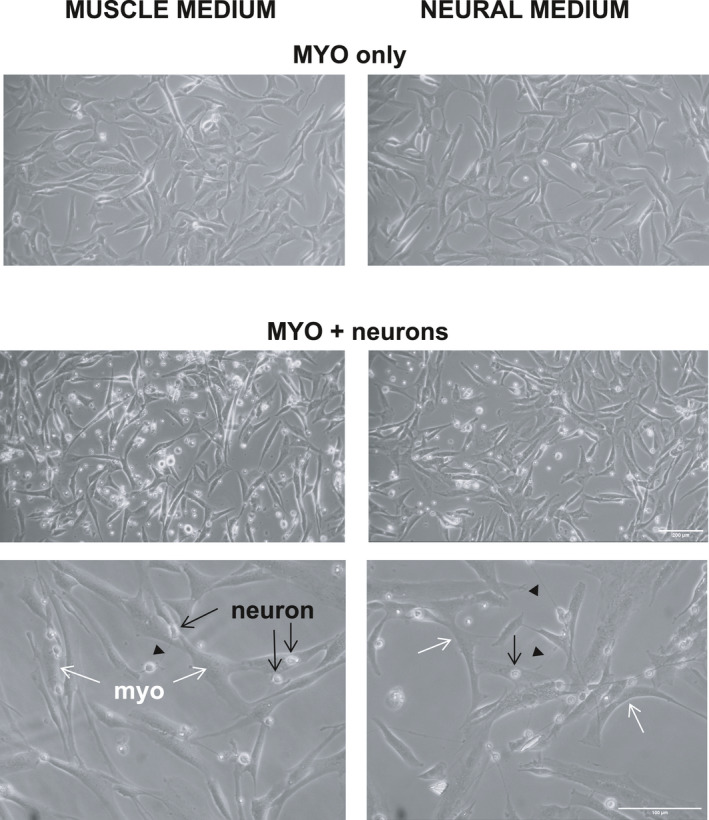
Human myogenic cell and rat neuron co‐cultures. Phase‐contrast microscopy images of human myogenic cells (myo; examples indicated by white arrows) cultured alone or together with rat neurons, in either muscle medium (left) or neural medium (right). In co‐cultures, neuron cell bodies (examples indicated by black arrows) can be seen on top of myo more often than on the glass surface between myo cells. Within 24 h of plating onto an established layer of human myo, activity of the neurons is evident from growth of neurites (examples indicated by black arrow heads), which are visible as thin threads connecting neuron cell bodies. Scale bars: 100 μm for the higher magnification images (last row) and 200 μm for the other (lower magnification) images

**FIGURE 4:**
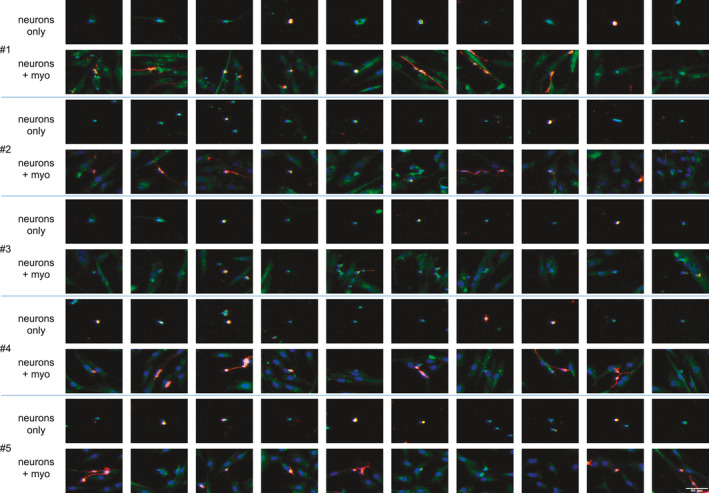
Influence of the presence of human myogenic cells on neurite outgrowth. Images represent 10 randomly chosen neural nuclei (blue, DAPI) for each of the 5 human myoblast donors (#1–5), in co‐culture (neurons + myo) or monoculture (neurons only). Neuronal neurites (red, GAP43) are only visible in the neurons + myo co‐cultures and not in the respective cultures of neurons only. Scale bar, 50 μm

The aBTX stain could not be distinguished from background, suggesting a low level of expression. As a general observation, after 24 h of co‐culturing, CGNs were observed on top of myogenic cells rather than attaching to the coverslip surface (Figure 3). Based on the evaluation of neurite outgrowth, neurites were only observed when neurons were cultured on a layer of human myogenic cells, compared to when cultured directly on the glass coverslip alone (Figure 4). This was the case for four out of five of the human myogenic cell cultures, with no neurite formation observed in either condition for myogenic cell culture #3.

### 3.3 Human myogenic cell gene expression

**FIGURE 5:**
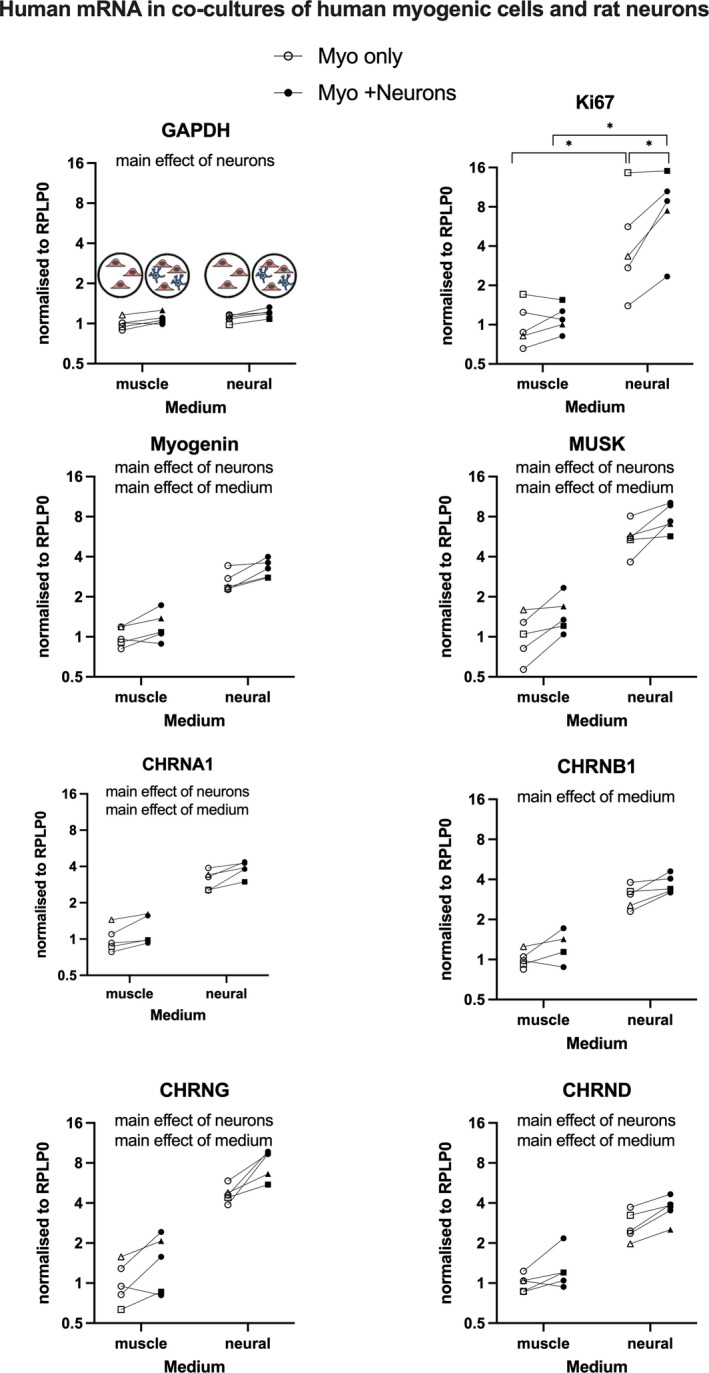
Human myogenic cell PCR data. Data are human mRNA and therefore specific for myogenic cells (Myo). Myo were cultured alone or with rat neurons (Myo + Neurons), in either muscle medium or neural medium. Data points displayed represent the five human myogenic cell donors. Data were analyzed by two‐way repeated measures ANOVA. Main effects (*p* < 0.05) and post hoc test outcomes (*p < 0.05) are indicated

As can be seen in Figure 5, gene expression levels of human myogenic cells, co‐cultured with rat CGNs, were generally higher compared to myogenic cells cultured alone, and generally higher in neural medium, compared to muscle medium. This was the case for myogenin, MuSK, CHRNA1 (AChR‐alpha), CHRND (AChR‐delta), and CHRNG (AChR‐gamma), where main effects of medium and neurons were detected. A main effect of medium only was detected for CHRNB1 (AChR‐beta). A main effect of neurons only was found for GAPDH. Ki67 was the only mRNA where a neuron–medium interaction was uncovered, revealing greater levels in the presence of neurons in neural medium but not in muscle medium. Levels of CHRNE (AChR‐epsilon) were below lower detection limits and are therefore not shown.

### 3.4 Rat neuron gene expression

**FIGURE 6:**
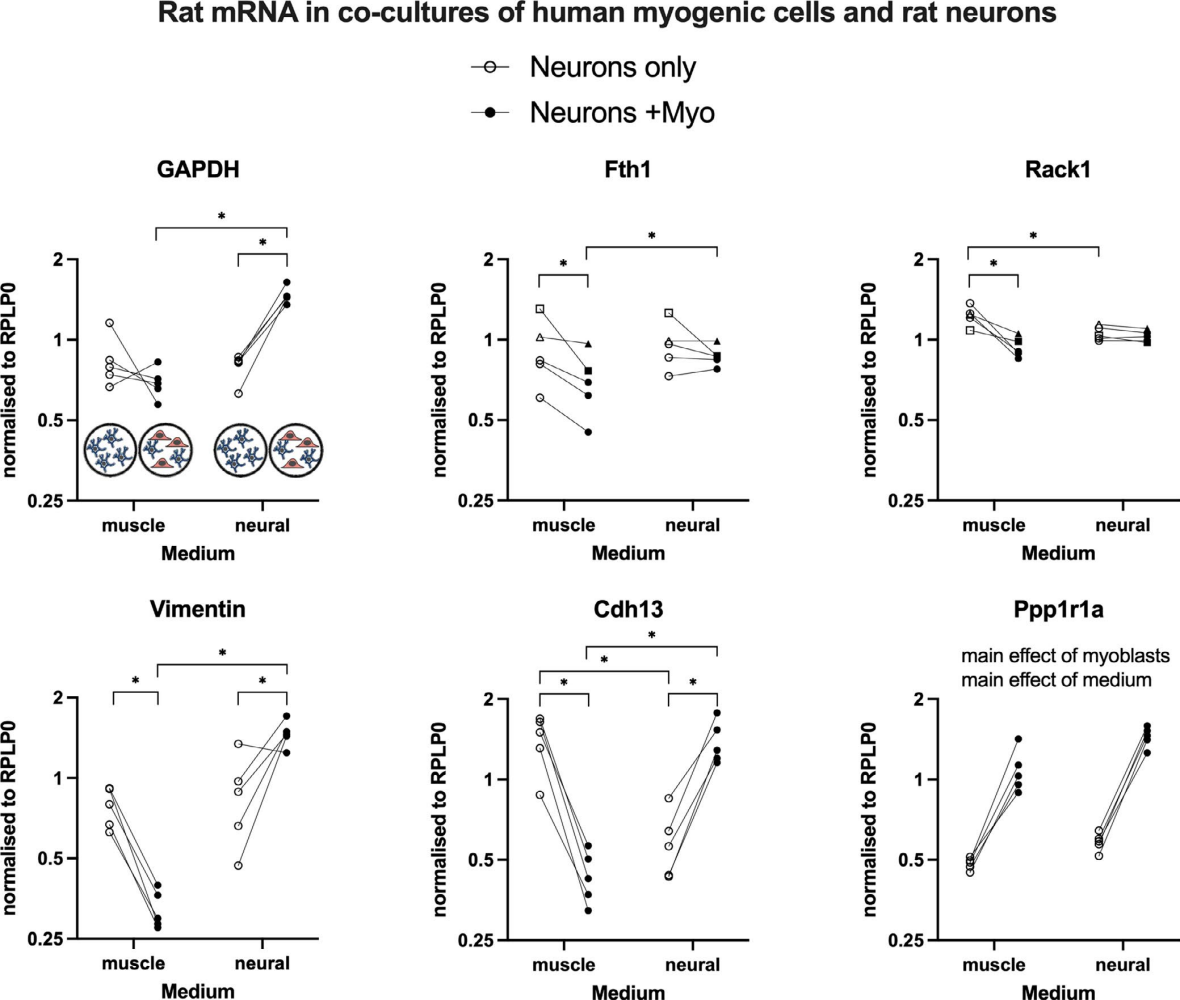
Rat neuron PCR data. Rat cerebellar granule neurons were cultured alone or with human myogenic cells (Neurons + Myo), in either muscle medium or neural medium. Data are rat mRNA and therefore specific for neurons. Data points displayed represent the five human myogenic cell donors and their neuron‐only monoculture controls. Data were analyzed by two‐way repeated measures ANOVA. Main effects (*p* < 0.05) and post hoc test outcomes (*p < 0.05) are indicated

The presence of human myogenic cells in cultures of rat CGNs clearly influenced CGN gene expression (Figure 6). The two‐way repeated measures ANOVA revealed interactions for five out of the six targets measured, with a strong influence of the type of medium and the presence of myogenic cells. Several distinct patterns were observed. In muscle medium, gene expression levels of Fth1, Rack1, vimentin, and Cdh13 were greater in neurons cultured alone than in the presence of myogenic cells. The reverse was seen for some mRNA targets in neural medium, with lower gene expression levels observed in mono‐cultures versus co‐cultures for GAPDH, vimentin, and Cdh13. Ppp1r1a demonstrated a unique pattern of clear main effects of medium type and myogenic cells, with lower values detected in mono‐cultures versus co‐cultures.

## 4 DISCUSSION

The main findings of this study are that the presence of rat cerebellar neurons strongly stimulates primary human myogenic cells in vitro, with upregulated transcription of key genes for cell proliferation, myogenic differentiation, and the AChR subunits. This influence was reciprocated in the neurons, where co‐culture with myogenic cells for 24 h lead to an enrichment of neural growth cone gene transcripts and neurite growth as assessed morphologically. These findings provide support for mutually beneficial bidirectional signaling between neural and myogenic cells.

To our knowledge, this is the first detailed analysis of primary human myogenic cells from multiple donors, co‐cultured with cerebellar neurons. An advantage of mixed species co‐cultures is being able to measure signaling in a species‐specific manner. Thus, in our mixed cell cultures containing human myogenic cells and rat neurons, human mRNA measurements reflect gene transcription in the myogenic cells alone. Here, a significant stimulatory effect of the presence of rat CGNs was detected for seven of the eight gene targets measured, indicative of greater cell metabolism (GAPDH), proliferation (Ki67), differentiation (myogenin), and myogenic activity important for NMJ formation (MuSK and AChRs). Three of the four AChR subunits (alpha‐1, gamma, and delta) demonstrated a similar strong upregulation in the presence of neurons, compared to myogenic cells cultured alone. The fact that the levels of the epsilon subunit were too low to detect is in line with this subunit not being produced until later stages of myogenesis, where it is reported to replace the gamma subunit (Cetin et al., 2020; Gu & Hall, 1988; Mishina et al., 1986; Missias et al., 1996).

The other striking, yet unexpected, finding was that the neural medium was associated with greater gene expression of most targets measured, when compared to the muscle medium. This could potentially be explained by the neural medium facilitating better conditions for the neurons, which in turn would provide a stronger stimulus to the myogenic cells. However, we also had controls where myogenic cells were cultured alone and the statistical analysis revealed a main effect of medium, indicating that myogenic cells in mono‐ or co‐culture were affected similarly by the type of medium. Ki67, a cell proliferation marker, was the exception to this, where only myogenic cells in co‐culture displayed higher gene expression levels than myogenic cells cultured alone, in neural medium. While the muscle medium is changed in conjunction with addition of neurons, to favor myogenic cell differentiation over proliferation, the neural medium is also serum‐free, exerting a similar anti‐proliferative effect to the muscle medium. The further elevation in Ki67 mRNA in the presence of neurons is surprising, since it was expected that neurons would push the myogenic cells toward maturity. Thus, the reason for the persistent myogenic cell Ki67 transcription in neural medium, and in co‐culture with neurons, is unclear. While the simultaneous observation of a stimulatory effect of neurons on genes regulating both myogenic cell proliferation and differentiation may seem paradoxical, it is possible that this reflects the presence of different populations of cells actively proliferating or differentiating at the time of analysis, or that this is a feature related to the cerebellar nature of the neurons, rather than motoneurons.

Taking all the myogenic cell PCR data together, it is clear that the presence of neurons provides a strong stimulatory effect on primary human myogenic cells in vitro, which is fully in line with the importance of neural input for myofiber survival in vivo and further underlines the value of neuron‐myoblast co‐cultures, as presented recently (Saini et al., 2019, 2020). Here, we contribute with novel quantitative data identifying specific genes that are regulated by this cell–cell interplay. Furthermore, it is interesting that neurons derived from the cerebellum appear to stimulate myogenic cells in a similar manner to what we would expect to see with motoneurons, suggesting common signaling pathways in neurons of cerebellar and spinal cord origin, at least in the first 24 h of co‐culture. Further work over a longer time course is required to investigate the potential of cerebellar neurons to establish NMJ contacts with myogenic cells.

In addition to signaling from the neurons to myogenic cells, there were also indications of a beneficial effect of the presence of myogenic cells on the neurons. For example, a general observation was that the neurons appeared to be preferentially located on top of the myogenic cells rather than on the surface of the glass coverslip. In addition, our qualitative analysis of immunocytochemistry images indicated that neurites were only observed in neurons co‐cultured with myogenic cells and not in neurons cultured alone. This is the first time this has been evaluated with primary human myogenic cells from several donors and at low passage number. In addition to the morphological analysis, PCR analysis of rat mRNA revealed greater GAPDH, vimentin, Cdh13, and Ppp1r1a in co‐culture, versus monoculture, in neural medium. Curiously, this picture was not mirrored for muscle medium, where co‐culture lead to lower levels of Fth1, Rack1, vimentin, and Cdh13 than monoculture. Ppp1r1a was the only target to display the same pattern in both medium types. The disparity between the myogenic cells and neurons with regard to the influence of medium type in mono‐ versus co‐culture is interesting and warrants further investigation. The selection of the panel of rat mRNA targets was based on a study comparing the subcellular transcriptomes of neuron growth cones versus their parent cell bodies (Poulopoulos et al., 2019), and therefore represents neurite outgrowth. In terms of the mechanism behind the stimulation of neurite outgrowth, this has been reported to be positively regulated by neural cell adhesion molecule (NCAM)‐expressing fibroblasts (Kohler et al., 2010), so it is possible that the NCAM expressed by human myoblasts and myotubes (Mackey & Kjaer, 2017) can stimulate neurite outgrowth in a similar manner. Indeed, NCAM is believed to be expressed by denervated myofibers in order to facilitate the contact of an axon growth cone with its target cell, potentially through binding to glial cell‐derived neurotrophic factor (GDNF) as suggested (Paratcha et al., 2003), which could explain the preferential location of the neurons on the myogenic cells. While requiring further study, our observations of a stimulatory influence of human myogenic cells on neurons in vitro support a capacity for human myogenic cells to attract and stimulate growth of cerebellar neural cells during a 24‐h period.

Taken together, the novel findings of this study provide support for reciprocal signaling between primary human myogenic cells and rat cerebellar neurons, in line with an active role for both the neuron and muscle cell in the formation of cell–cell contact, such as at the NMJ. However it is unlikely that cell signaling occurred via NMJ contacts in the current study, first because cerebellar neurons, and not motoneurons, were used and second because cells were analyzed after only 24 h of co‐culture [NMJ formation is reported to take up to 2 weeks (Guo et al., 2011; Saini et al., 2019, 2020)]. Future work could build on these findings to determine the nature of the communication between cerebellar neurons and myogenic cells, that is, whether it is through release of factors into the shared medium or through direct, transient, “kiss‐and‐run” mode of cell–cell contact. Both forms of contact are important, in signaling from the muscle to the brain via the circulation across the blood–brain barrier, and directly through the point of contact between muscle and motoneuron at the NMJ.

## CONFLICT OF INTEREST

None of the authors have any conflict of interest to declare.

## AUTHOR CONTRIBUTION

MT, CPP, PS, MRK, MK, and ALM designed the experiment; MT, SP, CS, CYCY, and JJ performed the cell culture experiments; MT and PS performed the analyses; MT, PS, MK, and ALM drafted the manuscript.
